# Diversity-sensitive measures in German hospitals – attitudes, implementation, and barriers according to administration managers

**DOI:** 10.1186/s12913-022-08058-3

**Published:** 2022-05-23

**Authors:** Fabian Erdsiek, Tuğba Aksakal, Maria Mader, Munzir Idris, Yüce Yılmaz-Aslan, Oliver Razum, Patrick Brzoska

**Affiliations:** 1 Witten/Herdecke University, Faculty of Health, School of Medicine, Health Services Research, Alfred-Herrhausen-Strasse 50, 58448 Witten, Germany; 2 Bielefeld University, School of Public Health, AG 3: Epidemiology and International Public Health, Bielefeld, Germany; 3 Bielefeld University, School of Public Health, AG 6: Health Services Research and Nursing Science, Bielefeld, Germany

**Keywords:** Diversity, Hospital care, Germany, Culturally sensitive

## Abstract

**Background:**

Populations have varying needs and expectations concerning health care that result from diversity characteristics such as a migrant background, gender identity, disability, and age. These needs and expectations must be considered to ensure adequate utilization and quality of health services. Approaches to address diversity do exist, however, little is known about the extent to which they are implemented by health care facilities. The present study aims to examine, which measures and structures hospitals in Germany employ to address diversity, as well as which barriers they encounter in doing so.

**Methods:**

A mixed-mode survey among administration managers of all registered German hospitals (excluding rehabilitation hospitals; *n* = 1125) was conducted between May and October 2019 using pen-and-paper and online questionnaires. Results were analyzed descriptively.

**Results:**

Data from *n* = 112 hospitals were available. While 57.1% of hospitals addressed diversity in their mission statement and 59.9% included diversity considerations in quality management, dedicated working groups and diversity commissioners were less prevalent (15.2% each). The majority of hospitals offered multi-lingual admission and exit interviews (59.8%), treatments or therapies (57.1%), but only few had multi-lingual meal plans (12.5%) and seminars or presentations (11.6%). While 41.1% of the hospitals offered treatment and/or nursing exclusively by staff of the same sex, only 17.0% offered group therapies for both sexes separately. According to the managers, the main barriers were a lack of financial resources (54.5%), a lack of incentives from the funding providers (49.1%), and organizational difficulties (45.5%). Other reported barriers were a lack of conviction of the necessity among decision makers (28.6%) and a lack of motivation among staff members (19.6%).

**Conclusions:**

Administration managers from only a small proportion of hospitals participated in our survey on diversity sensitivity. Even hospitals of those who did are currently not adequately addressing the diversity of staff members and patients. Most hospitals address diversity on an ideational level, practical measures are not widely implemented. Existing measures suggest that most hospitals have no overarching concept to address diversity in a broader sense. The main reported barriers relate to economic aspects, a lack of support in organizing and implementing corresponding measures and a lack of awareness or motivation.

**Supplementary Information:**

The online version contains supplementary material available at 10.1186/s12913-022-08058-3.

## Background

Germany’s population is becoming increasingly diverse in terms of age, gender, cultural/ethnical background, sexual preference and other characteristics. Many minorities are more and more able to voice their needs and expectations concerning health care. These needs and expectations vary between people with different diversity characteristics [1, 2]. Failing to address these needs and expectations can hamper access to and the effectiveness of health services.

Homophobia, discrimination and a lack of knowledge and awareness of the relationship between health or illness and gender identity are perceived as important barriers to adequate access to health care by gender diverse persons and/or individuals of non-heterosexual orientation [3, 4]. In Germany, LGBTI (lesbian, gay, bisexual, transgender and intersex) persons fear discrimination (including heteronormative assumptions) and report missing awareness among health professionals regarding psychosomatic problems related to stigmatization and a lack of contact points and adequate low-level counseling in health care settings [5]. Immigrants and ethnic minorities also show lower utilization of various health services [6–8]. They experience discrimination as well, but additionally report language barriers, limited cultural sensitivity of services and difficulties in finding information about existing health services [9]. Limited knowledge about existing services, legal rights to care and several aspects associated with the process of migration add to these experiences; examples are mistrust towards institutions, experience of persecution, or concerns about immigration status, which can impair utilization of health services [9]. When trying to access hospital care, individuals with intellectual disabilities, another vulnerable population group, can encounter a broad spectrum of barriers as well, including negative attitudes from hospital staff, limited knowledge among health professionals about special needs and communication requirements, and lack of support for professional carers or other accompanying persons [10, 11]. Other dimensions of diversity have similarly been associated with barriers or inequalities in utilizing specific health services, e.g., socioeconomic status [12] or homelessness [13].

In contrast to conceptual frameworks promoting cultural competency and responsiveness to diversity in a wider sense, strategies to address needs and expectations of diverse patients in German hospitals often focus on one or few specific groups, e.g., migrants or linguistically diverse individuals [14–19]. Such approaches centering on cultural diversity are inadequate to address diversity overall and specifically interactions (intersectionality) between different dimensions of diversity [20–22]. Currently, it is still largely unclear to what extent German hospitals have implemented measures and strategies to address diversity among patients and hospital staff, as well as which resources and barriers are relevant for hospitals in terms of introducing and maintaining such measures.

The present study aims to examine how hospital managers in Germany perceive the relevance of catering to diverse populations, which measures and strategies hospitals employ to address diversity, as well as which barriers they encounter in implementing and maintaining these approaches.

## Methods

For our study, we conducted a postal survey among administration managers of all acute care hospitals and other hospitals (*n* = 1125) listed in the German hospital registry [23], excluding rehabilitation hospitals. We contacted the respective managers between May and June 2019. Participants who did not respond in the first wave were invited again to participate between August and October 2019 using a pen-and-paper or, alternatively, an online questionnaire. Non-responders were invited to fill out an anonymous post card to indicate their reasons for not taking part in the survey. With this approach, we achieved a response rate of 10.3% (*n* = 112) and, in addition, received non-responder post cards from 6.3% (*n* = 71) of hospitals.

The questionnaire in use (Supplementary File 1) was developed specifically for this study and pretested through cognitive interviews. Most interviewees considered the questionnaire to be easily understandable and comprehensive. They provided some suggestions for minor modifications to enhance clarity which we implemented accordingly. Topics of the survey were the perceived relevance of a diversity-sensitive approach to health care, diversity-sensitive measures and strategies implemented by the hospital (pertaining to the diversity of both staff and patients), and perceived resources and barriers in implementing and maintaining diversity-sensitive health care in the respective hospital.

The data was analyzed descriptively using Stata 15.

For our analysis we included information from all 112 hospitals which completed the questionnaire. Hospitals which only replied by means of the non-responder postcard stated that reasons for not participating in the survey were lack of time (81.7%), the questionnaire considered being too long (16.9%), and respondents feeling the survey questions were not applicable to their facility (14.1%).

## Results

The majority of hospitals in our sample were non-profit (*n* = 58; 50.9%), while a smaller proportion were operated by public (n = 58; 23.7%) or private organizations (*n* = 24; 21.1%). Most hospitals employed staff of 500 persons or more and catered to 1000 or more patients per month (Table 1).

**Table 1:** Characteristics of German hospitals participating in the postal survey (2019; *n* = 112)

Hospital characteristics		%	n
Ownership	Non-profit	51.8	58
	Public	24.1	27
	Private	23.1	26
	n/a	0.9	1
	*Total*	*100.0*	*112*
Number of staff	Less than 100	9.8	11
	100 to 499	29.5	33
	500 or more	59.8	67
	n/a	0.9	1
	*Total*	*100.0*	*112*
Approx. number of patients per month	Less than 100	8.0	9
	100 to 499	19.6	22
	500 to 999	14.3	16
	1000 or more	57.2	64
	n/a	0.9	1
	*Total*	*100.0*	*112*

### Perceived importance of diversity-sensitive health care

While 93.7% of administration managers stated that their hospital sufficiently addressed the needs of their patients, only 69.6% considered a diversity-sensitive orientation necessary. In contrast to that, 72.3% of respondents thought that it was important for staff to take part in training courses pertaining to diversity issues. A future, more diversity-sensitive orientation of hospitals in general was perceived as important by 77.7% of respondents, while only 53.6% reported the existence of corresponding plans for their own hospital. The majority of respondents considered sensitivity to diversity an important factor for patient satisfaction (83.0%) and staff satisfaction (75.9%). It was also regarded an important factor for effective treatment by 78.6%. In contrast, only 46.4% considered a diversity-sensitive approach as an important resource to increase the numbers of patients.

### Addressing diversity on an organizational level

On an organizational level, more than half of the hospitals surveyed reported to address diversity ideationally e.g., in their mission statement (57.1%) or as a consideration in their quality management efforts (59.8%), for example when handling patient complaints (Table 2). For both statements, around 10.7% of administration managers reported that inclusion of diversity aspects was currently in planning. In contrast, less than a quarter of hospitals had implemented concrete measures on a structural level. 15.2% of hospitals had designated or hired a diversity commissioner and 4.5% were planning to designate such a position. Another 15.2% of administration managers reported to have implemented working groups addressing diversity, while 10.7% were planning to do so. About one fifth of hospitals offered or planned to offer regular training courses and consultations for staff that were meant to enhance sensitivity to diversity (22.3%, resp. 19.6%). While 22.3% of respondents reported specifically addressing diversity in their public relation work, 14.3% were planning to do so.

**Table 2:** Diversity-sensitive measures in participating German hospitals on an organizational level (2019, *n* = 112)

	Already implemented	Currently in planning	No	n/a
Sensitivity to diversity in the mission statement	57.1%	10.7%	31.3%	0.9%
Sensitivity to diversity in quality management/ quality control (e.g., in handling patient complaints)	59.8%	10.7%	28.6%	0.9%
Designating or hiring a diversity commissioner	15.2%	4.5%	15.2%	0.0%
Working groups addressing diversity	15.2%	10.7%	74.1%	0.0%
Regular training courses and consultations for staff to enhance sensitivity to diversity	22.3%	19.6%	55.4%	2.7%
Public relation work specifically addressing diversity	22.3%	14.3%	63.4%	0.0%

### Addressing the diversity of employees

In our study, almost 50% of administration managers reported to ensure that their staff has a minimum proportion of male or female employees or of staff with physical or mental disabilities. 27.7 and 24.1%, respectively, considered it important to have a minimum proportion of older-aged employees or of employees with a migrant background.

In terms of personnel policies, results were inconsistent (Table 3). While the majority of respondents stated that they intended to keep personnel diverse concerning a number of dimensions, this is only partially reflected in specific actions. In terms of recruiting diverse staff, 50.9% of hospitals targeted health personnel from other countries for recruitment, while only 33.0% published vacancies in ways to specifically reach people with disabilities. Specific services, e.g., mentoring programs, to facilitate the integration of new employees with special needs were reported by 41.1% of respondents. Specific leadership training to increase the proportion of women in leadership positions was offered by 38.4% of hospitals. While the majority of hospitals provide German courses for employees (58.2%), only 27.7% offer training courses in other languages.

**Table 3:** Aspects of personnel policy addressing diversity of staff in participating German hospitals (2019, *n* = 112)

	Strongly agree	Agree	Disagree	Strongly Disagree	n/a
We strive to keep our personnel diverse concerning age, gender, migrant background, or physical and mental disabilities	21.4%	45.5%	22.3%	9.8%	0.9%
We recruit employees in other countries in a targeted manner	25.9%	25.0%	10.7%	35.7%	2.7%
We publish vacancies on community networks and other sources to explicitly reach people with disabilities	11.6%	21.4%	33.9%	32.1%	0.9%
We offer specific services (e.g., mentoring) to facilitate integration of employees with special needs	16.1%	25.0%	39.3%	18.8%	0.9%
We increase the proportion of females in leadership positions through specific leadership training	8.9%	29.5%	33.9%	23.2%	4.5%
We improve German language skills of our employees through training courses	31.3%	26.8%	16.1%	25.0%	0.9%
We improve foreign language skills of our employees through training courses	9.8%	17.9%	28.6%	42.9%	0.9%

In terms of work-life balance, results also indicated no clear trend. Many or most respondents stated they offered flexible working hours (91.7%), additional (potentially unpaid) leave days for special demands, e.g., childcare or caring for ill relatives (66.1%), and the possibility of working from home (59.8%). Fewer hospitals offered job sharing (49.1%) and corporate childcare (39.3%).

### Addressing the diversity of patients

When dealing with diverse patient groups, one major aspect to be considered is language. While 64.3% of administration managers reported to provide consent forms in different languages, other special forms and information leaflets in different languages were only provided by 47.3% (Table 4). Only a small number of hospitals offered meal plans/menus in languages other than German (12.5%). Overall, written materials were most commonly available in English (24.3%), Turkish (20.0%), Russian (17.3%), Arabic (8.7%) and Polish (7.0%). Admission and exit interviews were also available in different languages in 59.8% of all surveyed hospitals, while 57.1% of respondents stated to offer treatments and therapies in different languages. Specific consultations, such as social counseling or nutrition counseling, were available in different languages in 39.3% of hospitals, while 11.6% offered corresponding non-German language trainings, seminars, or presentations. Most commonly, these services were available in English (29.2%), Russian (22.9%), Turkish (14.5%), Arabic (13.1%) and Polish (9.2%).

**Table 4:** Implemented measures to address the diversity of patients in terms of language in participating German hospitals (2019, *n* = 112)

	%	n
**Documents and other written materials in different languages**		
Consent forms	64.3	72
Special forms and information leaflets	47.3	53
Meal plans/menus	12.5	14
**Consultations/treatments in different languages**		
Admission and exit interviews	59.8	67
Treatments and therapies	57.1	64
Consultations (e.g., social counseling or nutrition counseling)	39.3	44
Trainings, seminars, or presentations	11.6	13
**Language-independent resources**		
Signs and guideposts	38.4	43
Information leaflets and other materials	32.1	36
Therapy plans	7.1	8
Meal plans	8.0	9

Although many hospitals reported offering consultations or treatments in languages other than German, only 23.2% regularly used professional language interpreters, while 52.7% used professionals in some cases. Most hospitals frequently used medical personnel (69.6%) or patients’ friends and relatives (46.4%) for translation. To a lesser degree, hospitals relied on non-medical personnel (18.8%) or other patients (3.6%). Another way of overcoming language barriers in health care delivery is the use of language-independent materials that mostly rely on photos, pictures and pictograms instead of text to convey information. Only a minority of administration managers reported to use such, most of which provided language-independent signs and guideposts (38.4%) or information leaflets and similar materials (32.1%). Fewer hospitals had language-independent therapy plans (7.1%) or menus (8.0%) available.

Other aspects of diversity that have been associated with different needs and expectations are cultural and gender aspects. Addressing these aspects in therapeutic and non-therapeutic settings is another important component of diversity-sensitive health care. While 57.1% of respondents stated that they offered a culturally sensitive menu selection, most hospitals did not provide other known services that cater to needs and expectations informed by gender or culture (Table 5). While 41.1% of hospitals offered the option to be treated by staff of the same sex, only 17.0% reported to offer group therapies for men and women separately. Diversity-sensitive learning opportunities were provided in 36.6% of hospitals and 38.4% offered the possibility to choose between different therapeutic alternatives. In terms of non-therapeutical services and resources, 45.5% of hospitals reported to provide neutrally decorated prayer rooms. While foreign language TV stations were available in 28.6% of hospitals, only 11.6% provided foreign language newspapers or magazines. Only one hospital offered flexible opening hours of the cafeteria, which could be used for example by Muslims adhering to fasting rules during Ramadan.

**Table 5:** Available diversity-sensitive services and resources in participating German hospitals (2019, *n* = 112)

	%	n
**Medical or therapeutical services**		
Option to be treated exclusively by staff of the same sex	41.1	46
Group therapies offered for both sexes separately	17.0	19
Diversity-sensitive courses and presentations (e.g., diabetes training adapted to culturally influenced illness perceptions)	36.6	41
Option to choose between different therapies	38.4	43
**Non-therapeutical services and resources**		
Neutrally decorated prayer room	45.5	51
Foreign language newspapers or magazines	11.6	13
Foreign language TV stations (not including English speaking stations)	28.6	32
Culturally sensitive menu selection (e.g., kosher or halal)	57.1	64
Flexible opening hours of the cafeteria (e.g., after dusk during Ramadan)	0.9	1

### Perceived barriers to implementing diversity-sensitive structures and measures

Administration managers reported financial concerns as well as problems in establishing corresponding organizational structures and measures as main barriers to implementing diversity-sensitive care (Table 6). A lack of financial resources was reported by most of the respondents (54.5%), with 49.1% also reporting a lack of incentives from the respective funding organizations. Organizational difficulties were perceived by 45.5% of hospitals, while 35.7% saw a lack of information on how to implement diversity-sensitive measures as an important barrier that made the implementation of diversity-sensitive care difficult. Fewer respondents reported a lack of perceived necessity of such measures among decision makers (28.6%) and/or a lack of motivation among staff members to implement them. Only 20 hospitals (17.8%) – varying in ownership and size – reported no barriers in implementing diversity-sensitive measures, while 8.0% reported not intending to implement any diversity-sensitive measures at all.

**Table 6:** Perceived barriers to implementing diversity-sensitive strategies and measures in participating German hospitals (2019, *n* = 112)

	%	n
Lack of financial resources	54.5	61
Lack of incentives from the funding provider (health insurance companies, etc.)	49.1	55
Organizational difficulties	45.5	51
Lack of information on how to implement diversity-sensitive measures	35.7	40
Not all decision makers are convinced of the necessity of diversity-sensitive measures	28.6	32
Lack of motivation among staff members to implement corresponding measures	19.6	22
No barriers	17.8	20
Not applicable, no intention to implement diversity-sensitive measures at all	8.0	9

## Discussion

Health care services which are sensitive to the diverse needs and expectations of users are a prerequisite to providing patient-centered care to the entire population. Different approaches are available for implementing such services. Germany is one of the countries where little is known about which measures and strategies are employed by health care providers to address diversity among patients and hospital staff, as well as which resources and barriers promote or hinder the introduction and maintenance of such measures and strategies. Our survey showed that hospital administrators in Germany largely struggle to address diversity comprehensively and often do not use specific measures catering to diverse populations at all. Similar results were previously reported from other countries like the United States of America and Israel [24, 25]. While a larger number of hospitals address diversity on an ideational level, e.g., in the mission statement, the implementation of corresponding measures and structures on an organizational level as well as in terms of actual services or materials is not as widespread. In addition, implemented measures and structures often only address one or few dimensions of diversity, disregarding the intersectionality of diversity aspects in terms of potential interactions or combinations of diversity characteristics among patients. Recent findings on diversity sensitivity of German rehabilitation facilities and nursing homes show similar trends and suggest a more general lack of structured or coordinated efforts to address diversity in German health care facilities [26].

Correspondingly, measures of personnel policy to address the diversity of employees and keeping staff diverse are implemented only in few hospitals. These measures mostly focus on recruitment of personnel from other countries and integration of new staff, as well as on enabling staff to cater to the majority population through German courses. In contrast to this, measures to reach potential employees with diverse gender identities or with physical or mental disabilities are implemented only by a smaller number of hospitals. Similarly, most hospitals reported to not offer any training programs for their staff promoting skills to better cater to diverse, specifically non-German speaking, patients. While these measures address diversity, the motivation of implementing these measures is unclear. In recent years, a shortage of nurses and other health professionals has led to increased efforts to recruit personnel from other countries, which could explain the results [27]. These findings could also explain, why the overall cultural competence of staff as well as their awareness with respect to other diversity characteristics have been found to be limited in previous research [28, 29]. Similar findings have been reported for German rehabilitation hospitals and nursing homes [26]. In addition, some international studies among hospital staff and health care workers have shown a distinct lack of competence in responding to the specific needs and expectations of diverse persons as well, including linguistically diverse individuals, immigrants and LGBT persons [30, 31].

Specific organizational structures to develop or implement measures addressing the diversity of patients were also only present in the minority of hospitals. Only a small number of hospitals reported having designated diversity commissioners or implemented dedicated working groups. Language interpreters, culturally and linguistically diverse medical personnel and linguistically adapted written materials are commonly used measures to cater to diverse patients’ needs in health care [19]. According to our findings, most hospitals only provide those services and materials in languages other than German which are directly related to medical services, e.g., consent forms, admission and exit interviews, or treatments and therapies. Other, particularly non-therapeutic, materials and services are mostly not available in different languages. This is especially true for trainings, seminars, and similar ways of teaching, as well as meal plans, newspapers, or magazines. Similarly, language independent resources are only provided by a minority of hospitals. In terms of communicating with patients who have a limited German-language proficiency, hospitals – similar to other health care facilities in Germany [18, 26] – often rely on medical staff and lay interpreters such as patients’ family members and friends. Since professional interpreters are not reimbursed by the statutory health insurance, the cost of these services may prevent especially smaller hospitals from using them. Unlike translation provided by trained interpreters, this however may be associated with limited translation quality and data protection concerns [32]. When addressing other aspects of diversity, such as gender and culture, only culturally sensitive menu selections (e.g. kosher or helal food) are available at the majority of hospitals. While some hospitals also offer further services and resources, findings suggest no clear underlying concept, but rather a patchwork of measures and services.

While many administration managers considered diversity responsiveness as an important issue for hospitals in general, far fewer see this as an important topic for their own hospitals. Similarly, many respondents consider diversity to be an important beneficial factor for better treatment outcomes, patient and staff satisfaction, though it is not regarded as an important factor for increasing patient numbers. This suggests a general awareness of the importance of addressing diversity in society, but a perceived low economic benefit for the individual hospital. Studies from other countries also suggest that more evidence on positive business effects may lead to intensified efforts and improve cultural competency [24, 25]. This is also supported by the finding in our study that a lack of incentives and financial resources are among the main reported barriers to introducing diversity-sensitive measures. Some administration managers also reported low perceived necessity to implement such measures and a lack of motivation to carry them out. In Germany, hospitals are funded from two sources: the government of the respective federal state and the health insurance providers. While the states are responsible for financing investments into the infrastructure, health insurance funds pay the costs of treatment. In this respect, providing adequate incentives would fall within the responsibility of the insurance funds as part of the negotiated payment for treatment costs. Since our findings only represent the perception of administration managers of hospitals, further evaluations are needed to determine the objective benefits of such incentives and the financial barriers hospitals encounter.

Apart from these aspects, hospitals often report a lack of competence and knowledge when trying to introduce or implement diversity-sensitive measures. Dedicated trainings, workshops, and guides can help to address these difficulties and to reduce initial obstacles. Only a small number of hospitals reported no barriers in implementing measures. In addition, there are still 8.0% of hospitals that do not intend to address diversity at all.

### Strengths and limitations

According to our knowledge, this study is the first to provide information on the sensitivity and responsiveness of German hospitals to the diversity of patients and staff on an organizational level. In addition, we identified several important barriers to implementing respective strategies and measures. These findings can inform future research focusing on the development and implementation of suitable concepts for diversity sensitive health care. Our study also has several limitations that need to be mentioned. For our study we relied on information provided by hospital administration managers. This only allows for a subjective account of how facilities are responding to diversity, which would need to be corroborated by standardized assessments. Therefore, in our study, information pertaining to the perception or motivation of employees and other decision makers, on which we also have no further information with respect to their age or sex, could be biased. The findings could either over- or underestimate the true level of diversity sensitivity in the facilities. For example, while almost all of the respondents stated their hospital addresses the needs of their patients, patients themselves could have a different impression. In addition, responses to other items concerning the implemented measures suggest, that the level of diversity awareness may be lower than perceived by the respondents. In contrast, specific measures taken by hospital staff without consultation of administration managers or measures not included in our questionnaire may have led to an underestimation of diversity sensitivity. To address the latter aspect, we included several open questions to identify such measures, which did not yield any valuable results. A more detailed, stratified analysis by the size or ownership of the hospitals, which would have been beneficial for the interpretation of the findings, was not possible due to the small sample size. Compared to other official statistics, hospitals from non-profit organizations were widely overrepresented in our sample. The low response rate and the structure of our sample indicate that our findings may not be representative for all hospitals in Germany. Similarly, administration managers with no interest in the topic or working at hospitals with no implemented measures to address diversity may be underrepresented, which may have led to an overestimation of diversity sensitivity in our study. This would suggest an even stronger necessity to increase awareness of the importance and the potential benefits of diversity-sensitive and responsive health care among hospital managers and other involved decision makers.

## Conclusions

This study is one of the first examining the extent to which German hospitals are sensitive or responsive to the needs and expectations of diverse patients and personnel from an organizational perspective. According to the responses of their administrators, the majority of hospitals in our study are currently not adequately addressing the needs of diverse staff members or patients. While a larger proportion of hospitals has implemented measures to treat patients with a different language, other dimensions of diversity, such as cultural and gender aspects, as well as non-therapeutical aspects of care, are not addressed by most hospitals. Similarly, efforts to address the diversity of hospital staff are only present in a minority of the hospitals and generally address only some aspects of diversity. To improve access to and quality of health care for diverse patients and employees, more studies specifically evaluating concepts and strategies to address diversity and identifying potential financial benefits and costs are necessary. These studies, e.g., by employing a qualitative methodology, could allow further insights into the diversity awareness of hospitals and contribute to understanding decision making processes with respect to why certain measures and strategies are implemented while others are not. Funding providers and policy makers play an important role in creating general conditions to help hospitals implement corresponding strategies and measures. In addition, further efforts to allocate additional resources and provide additional support, e.g., through organization consulting and practical guidelines, could help interested hospitals.

## Supplementary Information

**Additional file 1: Supplementary file 1**. Questionnaire: Implementation strategies of diversity sensitive healthcare. Survey of healthcare facilities. Self-developed questionnaire to research the state of diversity sensitivity – including awareness, strategies and measures, and perceived barriers – of German hospitals *(in German). (PDF 264 kb)*

## Data Availability

The dataset used in the study is available from the corresponding author upon reasonable request.

## Abbreviation

LGBTI: Lesbian gay bisexual transgender and intersex
