# Supplementary material for: Diversity-sensitive measures in German hospitals – attitudes, implementation, and barriers according to administration managers
Source: BMC Health Serv Res. 2022 May 23;22:689. doi: 10.1186/s12913-022-08058-3 (PMC9128136; doi:10.1186/s12913-022-08058-3)
Supplement: Supplementary file 1 — Additional file 1: Supplementary file 1. Questionnaire: Implementation strategies of diversity sensitive healthcare. Survey of healthcare facilities. Self-developed questionnaire to research the state of diversity sensitivity – including awareness, strategies and measures, and perceived barriers – of German hospitals (in German). (PDF 264 kb) [file 12913_2022_8058_MOESM1_ESM.pdf]

UNIVERSITÄT BIELEFELD

universität Witten/Herdecke

Fragebogen

Umsetzungsstrategien einer diversitätssensiblen Gesundheitsversorgung

Befragung von Versorgungseinrichtungen

Sehr geehrte Damen und Herren,

mit diesem Fragebogen werden diversitätssensible Instrumente und Maßnahmen von Versorgungseinrichtungen erfasst sowie Strategien zur Umsetzung solcher Maßnahmen ermittelt. Der Fragebogen kommt im Rahmen der Studie DiversityKAT zum Einsatz. Nähere Informationen dazu finden Sie im beiliegenden Anschreiben.

Zwei Begriffe, die im Fragebogen verwendet werden, möchten wir vorab erläutern:

– **Diversitätssensibilität:** Mit Diversitätssensibilität oder diversitätssensiblem Umgang ist die Berücksichtigung der Vielfalt von Mitarbeitern/innen oder Patienten/innen im Hinblick auf Faktoren wie z.B. Geschlecht, Alter, Kultur, Sprache und Behinderung gemeint.

– **Maßnahmen diversitätssensibler Versorgung:** Hiermit sind Angebote, Leistungen und Maßnahmen gemeint, die zu einem diversitätssensiblen Umgang mit Mitarbeitern/innen und/oder Patienten/innen beitragen. Hierzu zählt zum Beispiel, mehrsprachige Informationen vorzuhalten oder eine durchgehende Barrierefreiheit zu gewährleisten. Weitere Beispiele finden Sie in diesem Fragebogen.

Im Fragebogen bitten wir Sie, die Antworten/Aspekte, die für Ihre Einrichtung zutreffend sind, anzukreuzen. Wir würden uns freuen, wenn Sie jede Frage beantworten würden. Wenn Sie sich an einigen Stellen nicht sicher sind, reichen uns auch Ihre Einschätzungen. Fragen, die nicht zu Ihrer Einrichtung passen, können Sie einfach auslassen. Das Ausfüllen des Fragebogens nimmt ca. 10-15 Minuten in Anspruch. Sollten Sie Fragen zur Studie oder zum Ausfüllen des Fragebogens haben, stehen wir Ihnen sehr gerne zur Verfügung.

Für Ihre Unterstützung danken wir Ihnen herzlichst.

Block 1: Organisationsbezogene Merkmale und Maßnahmen

**1. Bitte geben Sie die Art der Trägerschaft Ihrer Einrichtung an:**		
☐ Staatlicher Träger	☐ Freigemeinnütziger Träger	☐ Privater Träger

**2. Bitte geben Sie an, wie viele Mitarbeiter/innen Ihre Einrichtung hat:**							
☐ 1-5	☐ 6-9	☐ 10-19	☐ 20-49	☐ 50-99	☐ 100-199	☐ 200-499	☐ 500 und mehr

**3. Bitte schätzen Sie, wie viele Menschen Ihre Einrichtung im Monat etwa betreut/behandelt:**				
☐ Unter 50	☐ 50-99	☐ 100-499	☐ 500-999	☐ 1000 oder mehr

**4. Welche der folgenden Aspekte zur Diversitätssensibilität wurden in Ihrer Einrichtung umgesetzt bzw. sind in Planung?**	Nein	In Planung	Bereits umgesetzt
Berücksichtigung diversitätssensibler Aspekte im Leitbild	☐	☐	☐
Berücksichtigung diversitätssensibler Aspekte im Qualitätsmanagement (z. B. bei der Bearbeitung von Patienten/innen-Beschwerden)	☐	☐	☐
Gezielte Öffentlichkeitsarbeit zum Thema Diversitätssensibilität	☐	☐	☐
Gründung von Arbeitsgruppen zum Thema Diversitätssensibilität	☐	☐	☐
Benennung oder Einstellung von Diversitätsbeauftragten	☐	☐	☐
Überprüfung des Erfolgs (Evaluation) diversitätssensibler Maßnahmen	☐	☐	☐
Regelmäßige Fortbildungen/Schulungen/Fachberatungen für Mitarbeiter/innen zur Förderung der Diversitätssensibilität	☐	☐	☐
Einbeziehung externer Dienstleister oder Organisationen (z.B. Beratung/Coaching) in die Planung oder Umsetzung diversitätssensibler Maßnahmen	☐	☐	☐
Sonstige, und zwar: ✐ ______________________________		☐	☐

4.1 Sofern alle oder einige der o.g. Aspekte zur Diversitätssensibilität umgesetzt werden bzw. in Planung sind, auf welchen Dimensionen von Diversität liegt dann hauptsächlich der Fokus? (Mehrfachantworten möglich.)

☐ Migrationshintergrund/Kultur/Religion ☐ Geschlecht ☐ Alter ☐ Behinderung ☐ Sexuelle Identität

☐ Sonstige, und zwar: ✐ ______________________________

Block 2: Umgang mit der Diversität von Mitarbeitern/innen

**5. Bei der Personalpolitik achten wir darauf, dass unser Personal einen Mindestanteil hat an:** (Mehrfachnennungen möglich.)	
☐ Menschen mit Migrationshintergrund	☐ älteren Mitarbeiter/innen
☐ Mitarbeiter/innen mit körperlichen oder geistigen Einschränkungen	☐ männlichen bzw. weiblichen Mitarbeiterinnen

**6. In der Personalpolitik achten wir auf folgende Aspekte.** (Bitte teilen Sie zu jeder Aussage mit, wie sehr sie zutrifft.)	Trifft gar nicht zu	Trifft eher nicht zu	Trifft eher zu	Trifft voll zu
Durch spezielle Beratungs- und Förderangebote erhöhen wir den Frauenanteil in Führungspositionen.	☐	☐	☐	☐
Durch Sprachkurse fördern wir Deutschkenntnisse unserer Mitarbeiter/innen.	☐	☐	☐	☐
Durch Sprachkurse fördern wir Fremdsprachenkenntnisse unserer Mitarbeiter/innen.	☐	☐	☐	☐
Wir werben gezielt Mitarbeiter/innen im Ausland an.	☐	☐	☐	☐
Wir achten darauf, dass unser Personal vielfältig in Bezug auf Alter, Geschlecht, Migrationshintergrund oder körperliche/ psychische Einschränkungen bleibt.	☐	☐	☐	☐
Wir schreiben vakante Stellen auch über Gemeindenetzwerke und andere Quellen aus, um explizit benachteiligte Menschen (z.B. mit Behinderungen) anzusprechen.	☐	☐	☐	☐
Wir haben Maßnahmen (z.B. Leitbilder) implementiert, um Mitarbeiter/innen und Patienten/innen unterschiedlicher sexueller Identität (z.B. Transgender etc.) anzusprechen.	☐	☐	☐	☐
Wir haben Angebote (z.B. Mentorenprogramme) um neuen Mitarbeitern/innen mit besonderen Bedarfen (z.B. Behinderungen) den Einstieg zu erleichtern.	☐	☐	☐	☐
Sonstiges, und zwar: ✎ ________________________________		☐	☐	☐

**7. Welche der folgenden Angebote stehen in Ihrer Einrichtung für Mitarbeiter/innen zur Verfügung?** (Mehrfachantworten möglich)	
☐ flexible Arbeitszeiten	☐ Betriebs-Kindertagesstätte
☐ Home-Office (Möglichkeit, zumindest z. T. von zuhause zu arbeiten)	☐ Bei Bedarf zusätzliche (ggf. unbezahlte) Urlaubstage (zur Kinderbetreuung, Pflege von Angehörigen etc.)
☐ Job-Sharing (zwei Mitarbeiter/innen teilen sich eine Stelle)	☐ andere, und zwar: ✎ ______________________

**8. Gibt es spezielle Beratungs- und Unterstützungsangebote (z. B. Angebote der betrieblichen Gesundheitsförderung, Medienkompetenzschulung etc.) für…?** (Mehrfachantworten möglich.)	
☐ Es gibt (bisher) keine solchen Angebote.	☐ Es besteht kein Bedarf an solchen Angeboten.
☐ ältere Mitarbeiter/innen	☐ jüngere Mitarbeiter/innen
☐ weibliche Mitarbeiterinnen	☐ männliche Mitarbeiter
☐ Mitarbeiter/innen mit körperlichen oder geistigen Einschränkungen	☐ Mitarbeiter/innen unterschiedlicher Religion/ Weltanschauung
☐ Mitarbeiter/innen mit Migrationshintergrund	☐ Sonstiges: ✎ ____________________________

Block 3: Umgang mit der Diversität von Patienten/innen

Information und Kommunikation

**9. Werden die folgenden Materialien in verschiedenen Sprachen angeboten?**	Nicht zutreffend	Nein	In Planung	Ja
Schriftliche Einverständniserklärungen	☐	☐	☐	☐
Schriftliche Form- und Merkblätter zum Aufenthalt	☐	☐	☐	☐
Internetseiten/Homepage	☐	☐	☐	☐
Speisepläne	☐	☐	☐	☐
Sonstige, und zwar: ✐___________________________			☐	☐

9.1 Falls Materialien mehrsprachig angeboten werden, bitte nennen Sie die häufigsten Sprachen hiervon:
(Mehrfachantworten möglich)

☐ Englisch ☐ Türkisch ☐ Russisch ☐ Polnisch ☐ Arabisch Sonstige: ✐__________________

**10. Werden die folgenden Leistungen Ihrer Einrichtung in verschiedenen Sprachen angeboten?**	Nicht zutreffend	Nein	In Planung	Ja
Aufnahme- oder Entlassungsgespräche	☐	☐	☐	☐
Beratungsgespräche (z.B. Sozialberatung oder Ernährungsberatung)	☐	☐	☐	☐
Behandlungen	☐	☐	☐	☐
Schulungen, Vorträge, Seminare (sofern zutreffend)	☐	☐	☐	☐

10.1 Falls Leistungen mehrsprachig angeboten werden, bitte nennen Sie die häufigsten Sprachen hiervon:
(Mehrfachantworten möglich)

☐ Englisch ☐ Türkisch ☐ Russisch ☐ Polnisch ☐ Arabisch Sonstige: ✐____________________

**11. Werden die folgenden Materialien/Formulare in Ihrer Einrichtung sprachunabhängig (Verwendung von Abbildungen, Piktogrammen) angeboten?**	Nicht zutreffend	Nein	In Planung	Ja
Informationsmaterial	☐	☐	☐	☐
Beschilderung oder Wegweiser	☐	☐	☐	☐
Therapiepläne	☐	☐	☐	☐
Speisepläne	☐	☐	☐	☐
Sonstige, und zwar: ✐___________________________			☐	☐

12. Inwieweit werden in Ihrer Einrichtung die folgenden Personen zum Dolmetschen/Übersetzen für Patienten/innen mit geringen Deutschkenntnissen hinzugezogen?	Nicht zutreffend	Gar nicht	Manchmal	Oft
Angehörige, Freunde oder Bekannte der Patienten/innen	☐	☐	☐	☐
Zimmernachbarn oder andere Patienten/innen der Einrichtung	☐	☐	☐	☐
Ärztliches, pflegerisches oder therapeutisches Personal mit gleicher Muttersprache	☐	☐	☐	☐
Sonstiges Personal (z.B. Reinigungspersonal) mit gleicher Muttersprache	☐	☐	☐	☐
Speziell ausgebildete professionelle Dolmetscher/innen	☐	☐	☐	☐
Sonstige, und zwar: ✏______________________________			☐	☐

13. Stehen die folgenden Leistungen/Materialien in Ihrer Einrichtung zur Verfügung, um Menschen mit besonderen Bedarfen (z.B. Einschränkungen) die Kommunikation zu erleichtern?	Nicht zutreffend	Nein	In Planung	Ja
Schriftdolmetscher/innen (Verschriftlichung des Gesprochenen)	☐	☐	☐	☐
Informationsmaterial/Speisepläne in Brailleschrift	☐	☐	☐	☐
Bei Bedarf können Gebärdendolmetscher/innen herangezogen werden	☐	☐	☐	☐
Mitarbeiter/innen erhalten eine Liste von Kollegen/innen mit Fremdsprachenkenntnissen	☐	☐	☐	☐
Sonstige, und zwar: ✏______________________________			☐	☐

Unterbringung, Versorgung und Verpflegung

14. Welche der folgenden <u>therapeutischen</u> Angebote – sofern zutreffend – können Patienten/innen in Ihrer Einrichtung nutzen, falls sie es wünschen? (Mehrfachantworten möglich.)

☐ Gruppentherapien (z.B. Schwimm- und Bewegungsangebote), die getrennt für beide Geschlechter angeboten werden	☐ Angepasste Schulungen und Informationsveranstaltungen (z.B. Diabetesschulungen, die kulturelle Krankheitsvorstellungen oder Speisegewohnheiten berücksichtigen)
☐ Möglichkeit, ausschließlich vom Personal des eigenen Geschlechtes behandelt zu werden	☐ Möglichkeit, zwischen unterschiedlichen Therapiealternativen zu wählen
☐ Keine der zuvor genannten Angebote	☐ Sonstiges, und zwar: ✏________________
☐ Die genannten Angebote treffen auf meine Einrichtung nicht zu	

15. Welche der folgenden <u>nicht-therapeutischen</u> Angebote – sofern zutreffend – stehen in Ihrer Einrichtung zur Verfügung? (Mehrfachantworten möglich.)

☐ Gebetsraum/neutral gestalteter Religionsraum	☐ Fremdsprachige Zeitschriften/Zeitungen
☐ Fremdsprachige Fernsehsender (ohne englische Sender)	☐ Menüauswahl, die kulturelle Bedürfnisse berücksichtigt (z.B. Angebot von Helal- und koscherer Kost)
☐ Geänderte Öffnungszeiten der Cafeteria (z.B. im Ramadan nach Sonnenuntergang)	☐ Sonstiges, und zwar: ✏________________
☐ Die genannten Angebote treffen auf meine Einrichtung nicht zu	

16. **Wie werden die Wünsche von Patienten/innen im Hinblick auf ihre Versorgung in Ihrer Einrichtung erhoben?** (Mehrfachantworten möglich.)	
☐ Es findet keine Erhebung statt	☐ Mündlich während des Aufnahme-/Anamnesegesprächs
☐ Patienten/innen füllen einen Fragebogen aus	☐ Anders, und zwar: ✎ ____________

17. **Inwieweit werden folgende Informationen Ihrer Patienten/innen routinemäßig (z.B. im Rahmen der Anamnese) erhoben und ausgewertet (z.B. zum Zwecke des Qualitätsmanagements)?**	Wird nicht erhoben	Wird erhoben	Wird erhoben und ausgewertet
Nationalität	☐	☐	☐
Muttersprache/Sprachkenntnisse (inkl. Deutschkenntnisse)	☐	☐	☐
Übersetzungsbedarf (bei nicht ausreichenden Deutschkenntnissen)	☐	☐	☐
Konfession	☐	☐	☐
Fähigkeiten zu lesen und zu schreiben	☐	☐	☐
Wertvorstellungen bzw. kulturspezifische Einstellungen (z.B. in Bezug auf Krankheit)	☐	☐	☐
Essgewohnheiten (z.B. vegetarische, koschere und Helal-Kost)	☐	☐	☐
Besondere Bedarfslagen (z. B. Bewegungseinschränkungen, Minderung des Seh- oder Hörvermögens)	☐	☐	☐
Sexuelle Identität (z.B. Transgender etc.)	☐	☐	☐
Familiäre Situation (z. B. minderjährige Kinder oder zu pflegende Angehörige)	☐	☐	☐
Sonstiges, und zwar: ✎ ____________		☐	☐
18. **Die ausgewerteten Daten werden dafür genutzt, um unsere therapeutischen und nicht-therapeutischen Angebote den Bedürfnissen der Patienten/innen anzupassen**	☐ ja ☐ nein ☐ in Planung		

Sonstige Aspekte der Diversitätssensibilität

19. **Wie wichtig ist Ihrer Meinung nach die Diversitätssensibilität einer Versorgungseinrichtung für …**	Vollkommen unwichtig	Eher unwichtig	Eher wichtig	Sehr wichtig
…die Zufriedenheit von Patienten/innen	☐	☐	☐	☐
…die Zufriedenheit von Mitarbeiter/innen (z.B. durch bessere Behandlungs- und Prozessabläufe)	☐	☐	☐	☐
…den Behandlungserfolg von Patienten/innen	☐	☐	☐	☐
…die Steigerung von Patienten/innen-Zahlen	☐	☐	☐	☐
Sonstiges, und zwar: ✎ ____________		☐	☐	☐

20. **Bitte geben Sie an, inwieweit Sie den folgenden Aussagen zustimmen.**	Stimme voll zu	Stimme eher zu	Stimme eher nicht zu	Stimme nicht zu
Es ist notwendig, unsere Einrichtung diversitätssensibel auszurichten.	☐	☐	☐	☐
Unsere Einrichtung geht auf Behandlungsbedürfnisse von Patienten/innen ein.	☐	☐	☐	☐
Zukünftig ist eine stärkere Ausrichtung unserer Einrichtung auf diversitätssensible Aspekte vorgesehen.	☐	☐	☐	☐

20. **Bitte geben Sie an, inwieweit Sie den folgenden Aussagen zustimmen.**	Stimme voll zu	Stimme eher zu	Stimme eher nicht zu	Stimme nicht zu
Fortsetzung				
Es ist wichtig, dass unsere Mitarbeiter/innen an Fortbildungen/Schulungen zum Umgang mit Diversität teilnehmen.	☐	☐	☐	☐
Eine diversitätssensible Ausrichtung ist aufgrund der derzeitigen finanziellen Situation für unsere Einrichtung nicht realisierbar.	☐	☐	☐	☐
Eine diversitätssensible Ausrichtung wird für viele Einrichtungen zukünftig eine noch größere Rolle als heute spielen.	☐	☐	☐	☐
Praxisnahe Handlungsempfehlungen könnten die Implementierung von diversitätssensiblen Maßnahmen fördern.	☐	☐	☐	☐

21. **Welche Hindernisse sehen/sahen Sie bei der Umsetzung einer diversitätssensiblen Versorgung in Ihrer Einrichtung?** (Mehrfachantworten möglich.)	
☐ Es gab/gibt keine Hindernisse	☐ Nicht zutreffend, da keine Absicht zur Implementierung solcher Maßnahmen in unserer Einrichtung besteht
☐ Mangelnde Motivation zur Umsetzung solcher Maßnahmen auf Seiten des Personals	☐ Nicht alle Entscheidungsträger/innen der Einrichtung sind von der Notwendigkeit solcher Maßnahmen überzeugt
☐ Organisatorische Schwierigkeiten	☐ Fehlende finanzielle Ressourcen
☐ Fehlende Anreize des Versorgungsträgers (Rentenversicherung, Krankenkassen etc.)	☐ Unklarheit, wie diversitätssensible Maßnahmen umgesetzt werden können
Sonstiges, und zwar: ✎ ________________________________	

22. **Woraus sollten Handlungsempfehlungen zur Implementierung diversitätssensibler Maßnahmen inhaltlich bestehen?** (Mehrfachantworten möglich.)		
☐ Fallbeispiele	☐ Hinweise zu weiterführenden Informationen	☐ Beispielmaßnahmen
☐ Hinweise zu Internetmaterialien	☐ Hinweise zum Umgang bei Implementierungsschwierigkeiten	☐ Anleitung zur Umsetzung von Maßnahmen/Konzepten
Sonstiges, und zwar: ✎ ________________________________		

23. **Wie würden Sie gerne informiert werden?** (Mehrfachantworten möglich.)			
☐ Arbeitsbuch/Broschüre	☐ Schulung/Seminar	☐ Internetseite	☐ Smartphone-App
Sonstiges, und zwar: ✎ ________________________________			

Falls Sie weitere Anmerkungen zum Forschungsthema haben, dann notieren Sie diese gerne hier: ✎

Herzlichen Dank für Ihre Teilnahme!

Bitte schicken Sie den ausgefüllten Fragebogen im beiliegenden Umschlag bis zum **07.06.2019** direkt an XXX. Das Porto übernehmen wir.
